# Fogarty Balloon-Assisted Flow Augmentation Combined With Prostaglandin E1 Versus Prostaglandin E1 Alone for No-Option Below-Knee Chronic Limb-Threatening Ischemia in a Resource-Limited Setting: A Prospective Quasi-experimental Study

**DOI:** 10.7759/cureus.114920

**Published:** 2026-08-21

**Authors:** Manjurul Hasan, Shoaeb Imtiaz Alam, Mufrid Kashem, Istiaq Ahmed

**Affiliations:** 1 Vascular Surgery, Dhaka Medical College and Hospital, Dhaka, BGD; 2 Cardiac Surgery, Dhaka Medical College and Hospital, Dhaka, BGD

**Keywords:** below-knee arterial disease, chronic limb-threatening ischemia (clti), fogarty balloon catheter, prostaglandin e1, resource-limited settings

## Abstract

Background

No-option chronic limb-threatening ischemia (CLTI) represents a challenging clinical condition in which conventional endovascular or surgical revascularization may not be feasible. In resource-limited settings, treatment options for patients with unreconstructable below-knee disease are particularly restricted. This study evaluated the clinical outcomes of a Fogarty balloon-assisted flow-augmentation technique combined with intravenous prostaglandin E1 (PGE1) compared with PGE1 alone in patients with no-option below-knee CLTI.

Methods

This prospective, single-center, quasi-experimental study was conducted at Dhaka Medical College and Hospital, Bangladesh, between January and December 2025 and included 100 patients with no-option below-knee CLTI. Patients received Fogarty balloon-assisted flow augmentation plus intravenous PGE1 (n = 50) or PGE1 alone (n = 50) through non-randomized allocation influenced by operating-room and surgeon availability and patient preference. The primary outcomes were six-month limb salvage and major amputation. Secondary outcomes included improvement in Rutherford clinical category, Visual Analog Scale (VAS) pain scores, complete ulcer healing, and all-cause mortality. Patients were followed for six months. The Fogarty technique was performed through an open femoral approach using a low-profile catheter with minimal balloon inflation and was not intended to replicate conventional high-pressure endovascular balloon angioplasty.

Results

Technical completion of the Fogarty procedure was achieved in 46 of 50 patients (92.0%). At six months, limb salvage was achieved in 82.0% of patients in the intervention group compared with 60.0% in the PGE1-alone group (*p* = 0.015), while major amputation occurred in 18.0% and 40.0%, respectively (*p* = 0.015). Improvement of at least one Rutherford clinical category occurred in 80.0% versus 56.0% of patients, respectively (*p* = 0.010). Among patients with baseline ischemic ulcers, complete ulcer healing occurred in 34 of 41 patients (82.9%) in the intervention group compared with 22 of 34 patients (64.7%) in the PGE1-alone group; this difference was not statistically significant (*p* = 0.071). Mean VAS pain scores were significantly lower in the intervention group at one, three, and six months (all *p* < 0.001). Six-month all-cause mortality was comparable between groups (4.0% vs. 6.0%; *p* = 0.65).

Conclusion

In this prospective quasi-experimental study, Fogarty balloon-assisted flow augmentation plus intravenous PGE1 was associated with higher limb salvage, lower major amputation, and lower VAS pain scores than PGE1 alone. Complete ulcer healing was numerically higher but not statistically significant. Given the non-randomized, single-center design and lack of routine objective perfusion assessment, these findings are preliminary, and the technique should be considered an exploratory salvage approach rather than a substitute for established revascularization.

## Introduction

Peripheral artery disease (PAD) is a progressive manifestation of systemic atherosclerosis characterized by stenosis or occlusion of arteries supplying the lower extremities. It affects more than 230 million people worldwide and is associated with substantial morbidity, impaired quality of life, cardiovascular events, and premature mortality [1]. The global burden of PAD continues to increase because of aging populations and the growing prevalence of diabetes mellitus, hypertension, chronic kidney disease, dyslipidemia, and tobacco use, particularly in low- and middle-income countries where access to specialized vascular care remains limited [2].

Chronic limb-threatening ischemia (CLTI) represents the most advanced stage of PAD and is characterized by ischemic rest pain, non-healing ulcers, or gangrene caused by severe arterial insufficiency. Contemporary international guidelines recommend prompt revascularization by endovascular intervention or surgical bypass whenever anatomically feasible, as restoration of blood flow remains the cornerstone of limb salvage [3,4]. However, a substantial proportion of patients have diffuse infrapopliteal disease, extensive arterial calcification, poor distal runoff, or lack suitable distal bypass targets, rendering them unsuitable for conventional revascularization. These patients are commonly classified as having no-option CLTI and continue to experience high rates of major amputation despite optimal medical therapy [5,6].

The angiosome concept has substantially improved understanding of lower-limb perfusion and wound healing. The foot and ankle are supplied by three major source arteries that perfuse six distinct angiosomes interconnected by an extensive collateral network of arterial-arterial connections known as choke vessels [7]. Direct revascularization of the artery supplying the affected angiosome generally provides the best wound-healing outcomes. Nevertheless, numerous clinical studies have demonstrated that satisfactory limb salvage can also be achieved through indirect revascularization, in which perfusion is restored through collateral channels and choke vessels when direct revascularization is not feasible [8,9]. The effectiveness of indirect revascularization depends largely on the integrity and functional capacity of these collateral networks, which may enlarge and remodel in response to increased blood flow and pressure gradients [10,11].

In patients with no-option CLTI, particularly in resource-limited settings, access to advanced revascularization procedures may be constrained by limited availability of specialized vascular services, endovascular facilities, and healthcare resources. In such circumstances, patients may be managed with medical therapy, wound care, analgesia, and, when ischemia progresses, amputation [12]. The development of simple, affordable, and reproducible adjunctive approaches for carefully selected no-option patients therefore represents an area of potential clinical interest. Such approaches, however, should be considered complementary or salvage strategies and should not be regarded as substitutes for established endovascular or surgical revascularization when those options are feasible. 

Prostaglandin E1 (alprostadil) has been used as adjunctive therapy in patients with no-option CLTI because of its vasodilatory, antiplatelet, anti-inflammatory, and microcirculatory effects [13]. Although prostaglandin therapy may improve tissue oxygenation, reduce ischemic rest pain, and promote ulcer healing, it does not restore large-vessel patency. Therefore, combining pharmacological optimization of the microcirculation with techniques that may enhance macrocirculatory inflow represents a biologically plausible strategy for improving tissue perfusion.

The Fogarty catheter was originally developed as a surgical device for arterial embolectomy and thrombectomy. In the present study, a low-profile Fogarty catheter was used through an open femoral arterial approach as an exploratory Fogarty Balloon-Assisted Flow-Augmentation technique. Importantly, this technique was not designed or intended to reproduce conventional high-pressure endovascular balloon angioplasty. The study does not assume that a Fogarty catheter can mechanically dilate a chronically occluded below-knee artery to the extent achieved by a dedicated angioplasty balloon. Instead, the clinical hypothesis was that carefully controlled catheter passage in selected patients might be associated with changes in distal perfusion through residual arterial channels and collateral pathways when conventional revascularization was not feasible. Whether such a maneuver produces measurable anatomical or physiological changes in arterial perfusion remains uncertain and requires objective imaging and physiological validation.

Despite the clinical need, evidence regarding such resource-adapted surgical approaches in patients with no-option below-knee CLTI remains limited. In particular, there is a lack of comparative clinical data evaluating a Fogarty Balloon-Assisted Flow-Augmentation Technique combined with PGE1 against PGE1 alone. Because the present study was conducted without randomization and without routine post-procedural angiographic or physiological confirmation of arterial changes, its purpose was not to establish anatomical recanalization or definitive treatment efficacy, but to provide preliminary clinical evidence that could inform future investigation.

Therefore, this study aimed to compare the six-month clinical outcomes of a Fogarty balloon-assisted flow augmentation combined with intravenous PGE1 versus intravenous PGE1 alone in carefully selected patients with no-option below-knee CLTI managed in a resource-limited setting. The primary outcomes were six-month limb salvage and major amputation. Secondary outcomes included ischemic rest-pain reduction, improvement in Rutherford clinical category, and ulcer healing. We hypothesized that the addition of the investigational technique to PGE1 would be associated with improved clinical outcomes compared with PGE1 alone.

## Materials and methods

Study design and setting

This prospective, single-center, quasi-experimental comparative study was conducted in the Department of Vascular Surgery at Dhaka Medical College and Hospital, a tertiary care teaching hospital in Bangladesh, between January 2025 and December 2025. The study evaluated whether the addition of a Fogarty balloon-assisted flow-augmentation technique to intravenous prostaglandin E1 (PGE1; alprostadil) was associated with improved clinical outcomes compared with PGE1 therapy alone in carefully selected patients with no-option below-knee CLTI.

During the study period, computed tomography angiography (CTA) was available for preoperative anatomical assessment and evaluation of revascularization options; however, dedicated therapeutic endovascular facilities for below-knee angioplasty or stent implantation were unavailable at the institution. Patients considered unsuitable for surgical bypass because of diffuse infrapopliteal disease, poor distal runoff, or absence of a suitable distal target vessel were therefore managed with either the investigational surgical technique combined with PGE1 or PGE1 alone.

The Fogarty balloon-assisted flow-augmentation technique was conceived and developed by Dr. Manjurul Hasan, resident surgeon and principal investigator, who also supervised the procedures performed during the study. Because this was a novel surgical modification without previous comparative clinical evaluation, the study was designed as an exploratory, hypothesis-generating investigation rather than a definitive efficacy trial.

The study protocol was approved by the Institutional Review Board of Dhaka Medical College and Hospital (ERC-DMC/ECC/121/2025), and written informed consent was obtained from all participants before enrollment. The study was conducted in accordance with the ethical principles of the Declaration of Helsinki.

Study population

Consecutive adult patients aged 18 years or older presenting with CLTI corresponding to Rutherford clinical categories 4-6 were screened for eligibility. The diagnosis of CLTI and no-option CLTI was established according to the 2019 Global Vascular Guidelines [14]. No-option CLTI was defined as CLTI in which conventional surgical or endovascular revascularization was considered technically infeasible or unavailable after multidisciplinary vascular assessment, based on the patient's clinical status and preoperative CTA findings. Patients were considered to have no-option CLTI when preoperative diagnostic angiographic evaluation demonstrated diffuse infrapopliteal arterial disease associated with poor distal runoff, severe tibial arterial calcification, long-segment occlusive disease, or absence of a suitable distal target vessel for bypass surgery. Because therapeutic below-knee endovascular intervention was unavailable at the study institution during the study period, balloon angioplasty and stent implantation could not be offered. All available angiographic findings were independently reviewed by two consultant vascular surgeons, and treatment decisions were reached through multidisciplinary vascular team discussion. Eligible patients had persistent ischemic rest pain and/or ischemic ulceration despite receiving guideline-directed medical therapy and were considered unsuitable for conventional surgical revascularization.

Patients were excluded if they had acute limb ischemia of less than 14 days' duration, embolic arterial occlusion, previous ipsilateral major amputation, popliteal artery aneurysm, active systemic sepsis, New York Heart Association class IV heart failure, end-stage renal disease requiring dialysis, pregnancy, known hypersensitivity to prostaglandin E1, or an anticipated life expectancy of less than six months because of advanced malignancy or terminal illness.

Sample size and group allocation

A total of 100 consecutive eligible patients were prospectively enrolled during the study period. Because no previous clinical study evaluating the Fogarty balloon-assisted flow-augmentation technique in patients with no-option CLTI was available, a formal sample-size calculation based on an anticipated treatment effect was not considered feasible. The sample size was therefore determined pragmatically according to the number of eligible patients presenting during the predefined study period. The study was intended to generate preliminary clinical evidence for subsequent adequately powered studies.

Randomization was not performed because of the quasi-experimental study design. Eligible patients who consented to undergo the investigational Fogarty balloon-assisted flow-augmentation procedure received the intervention. Eligible patients who did not undergo the investigational procedure because of operating-room availability, surgeon availability, or personal preference received PGE1 alone and constituted the comparison group. Fifty patients underwent the Fogarty balloon-assisted flow-augmentation procedure followed by intravenous PGE1 therapy, whereas 50 patients received intravenous PGE1 therapy alone.

The same inclusion and exclusion criteria were applied to both groups, and standardized medical and wound care was provided irrespective of treatment allocation. Baseline demographic and clinical characteristics were compared descriptively between groups to assess measured baseline differences. Because treatment assignment was non-randomized, the possibility of selection bias and residual confounding could not be eliminated.

Fogarty balloon-assisted flow-augmentation technique

The Fogarty balloon-assisted flow-augmentation technique was developed by Dr. Manjurul Hasan, who also supervised all procedures performed during the study. Unlike conventional use of the Fogarty catheter for arterial embolectomy or thrombectomy, this technique was designed as an exploratory mechanical maneuver intended to improve distal limb perfusion in selected patients with no-option CLTI. A commercially available 2F or 3F Fogarty® arterial embolectomy catheter (Edwards Lifesciences LLC, Irvine, California, USA) was used in all procedures. The Fogarty catheter is a well-established surgical device originally developed for arterial embolectomy and thrombectomy [15]. In the present study, it was used in an investigational manner and was not intended to perform conventional high-pressure balloon angioplasty. Minimal balloon inflation was used to facilitate gentle mechanical passage through chronically narrowed infrapopliteal arterial segments in an attempt to improve distal blood flow. The precise mechanism of action remains hypothetical and requires further experimental validation.

All procedures were performed under regional anesthesia. After systemic anticoagulation with intravenous unfractionated heparin (100 IU/kg), the common femoral artery was exposed through a standard groin incision. A transverse arteriotomy was performed, and the 2F or 3F Fogarty balloon catheter was carefully advanced into the infrapopliteal arterial system under tactile guidance.

The balloon was minimally inflated with sterile saline to maintain gentle contact with the arterial wall and was slowly withdrawn while avoiding excessive traction. Up to three gentle passes were performed according to tactile resistance encountered during the procedure. No high-pressure balloon inflation was used, and no attempt was made to reproduce conventional endovascular balloon angioplasty. Fluoroscopic guidance was not used because the procedure was performed as an open surgical maneuver in a setting without dedicated therapeutic endovascular facilities. Following catheter removal, the arterial lumen was irrigated with heparinized saline (1 IU/mL normal saline), and the arteriotomy was repaired using standard vascular surgical techniques. Distal limb perfusion was assessed intraoperatively using distal back bleeding or retrograde flow from distal arteries.

The purpose of the maneuver was to explore whether controlled passage of a low-profile Fogarty catheter could be associated with improved distal clinical perfusion through residual arterial channels and collateral pathways. The study did not assume or demonstrate that the procedure produced anatomical recanalization or luminal dilatation equivalent to conventional angioplasty.

Definition of procedural technical completion

Procedural technical completion was defined as successful passage of the Fogarty catheter through the intended arterial segment with restoration or presence of satisfactory distal back bleeding or retrograde flow at completion. This operational definition reflected completion of the surgical maneuver and intraoperative distal perfusion assessment and did not constitute evidence of arterial recanalization, luminal dilatation, or restoration of anatomical patency.

Prostaglandin E1 therapy

Patients in both groups received intravenous prostaglandin E1 (PGE1; alprostadil) according to the institutional treatment protocol. Each treatment cycle consisted of a total dose of 500 μg, administered as 166.67 μg daily for three consecutive days, with six treatment cycles repeated at approximately four-week intervals. This regimen represented the institutional protocol used during the study period. Published studies have evaluated intravenous alprostadil using different dosing regimens in patients with severe peripheral arterial disease and critical limb ischemia [13]. Therefore, the present dosing schedule should be regarded as an institution-specific protocol rather than a universally established CLTI regimen.

Concomitant medical management

All participants received standardized guideline-directed medical therapy throughout the study period irrespective of treatment allocation. This included dual antiplatelet therapy with aspirin and clopidogrel, high-intensity statin therapy, optimization of glycemic control and hypertension, smoking cessation counseling, meticulous wound care, culture-directed antibiotic therapy whenever clinically indicated, and analgesics according to pain severity. Diabetic foot care and ulcer management were provided in accordance with institutional treatment protocols.

Outcome measures

The primary clinical outcomes were six-month limb salvage and major amputation.

Secondary outcomes included improvement in Rutherford clinical category, minor amputation, repeat vascular intervention, complete ulcer healing, procedure-related complications, hospital readmission, duration of hospital stay, and all-cause mortality during the six-month follow-up period.

Limb salvage was defined as freedom from major amputation, defined as amputation at or above the ankle, during the six-month follow-up period. Ischemic rest pain was assessed using a 10-point Visual Analog Scale (VAS), with pain reduction evaluated by changes in VAS scores from baseline to subsequent follow-up visits. Between-group comparisons of VAS scores were performed separately at each prespecified follow-up time point. Ulcer healing was assessed by serial clinical examination and classified as complete healing, partial healing, or failure to heal. Complete healing was defined as complete epithelialization of the index ischemic ulcer without drainage or the need for further surgical wound intervention.

Follow-up

Patients were followed according to recommendations of the Global Vascular Guidelines for CLTI [14]. At each follow-up visit, comprehensive clinical assessment was performed, including evaluation of ischemic rest pain using the Visual Analog Scale, wound examination, documentation of ulcer healing, Rutherford clinical staging, handheld Doppler assessment of distal arterial signals, assessment of limb viability, and documentation of any repeat intervention, hospital readmission, amputation, procedure-related complication, or death. Patients unable to attend scheduled outpatient visits were contacted by telephone, and clinical information was obtained through local healthcare providers whenever feasible using standardized follow-up forms.

Data collection

Baseline demographic and clinical characteristics were prospectively recorded using standardized case record forms. Variables included age, sex, body mass index, diabetes mellitus, hypertension, dyslipidemia, smoking status, chronic kidney disease, coronary artery disease, previous cerebrovascular disease, duration of symptoms, Rutherford clinical category, ulcer characteristics, and angiographic findings. Operative details, perioperative variables, postoperative outcomes, and follow-up data were prospectively documented. To ensure data quality and consistency, all study records were independently verified by two investigators before statistical analysis.

Statistical analysis

Continuous variables were expressed as mean ± standard deviation or median with interquartile range according to data distribution, whereas categorical variables were presented as frequencies and percentages. Continuous variables were compared using the independent-samples Student's t-test or Mann-Whitney U test, as appropriate, while categorical variables were analyzed using the Pearson chi-square test or Fisher's exact test, as appropriate. Between-group comparisons of VAS pain scores were performed separately at baseline and at one, three, and six months using the independent-samples Student's t-test. Statistical analyses were conducted using IBM SPSS Statistics version 27.0 (IBM Corp., Armonk, NY, USA), and a two-tailed p-value of less than 0.05 was considered statistically significant.

## Results

A total of 100 consecutive eligible patients with no-option below-knee CLTI were included, with 50 patients assigned to the Fogarty balloon-assisted flow augmentation plus prostaglandin E1 (PGE1) group and 50 patients treated with PGE1 alone. All patients were followed for up to six months or until death, and complete outcome ascertainment was achieved.

The mean age was 63.8 ± 8.4 years in the Fogarty balloon-assisted flow augmentation plus PGE1 group and 64.5 ± 9.1 years in the PGE1-alone group (p = 0.71). Men accounted for 74.0% and 70.0% of the patients, respectively (p = 0.65). Diabetes mellitus was present in 78.0% of the patients in the intervention group and 80.0% in the control group (p = 0.81). Hypertension, dyslipidemia, current smoking, chronic kidney disease, and coronary artery disease were also comparable between groups, with no statistically significant differences.

Rutherford category 5 disease was present in 35 (70.0%) patients in the intervention group and 34 (68.0%) patients in the control group, while Rutherford category 6 disease was present in 15 (30.0%) and 16 (32.0%) patients, respectively (p = 0.83). Baseline VAS pain scores were similar between groups (8.4 ± 1.0 vs. 8.2 ± 1.1; p = 0.38). Overall, no statistically significant differences were identified in the measured baseline demographic or clinical characteristics between the two groups (Table 1).

**Table 1:** Baseline demographic and clinical characteristics of patients with no-option below-knee chronic limb-threatening ischemia (n=100) Data are presented as mean ± standard deviation (SD) for continuous variables and number (percentage) for categorical variables. Continuous variables were compared using the independent-samples Student's t test, and categorical variables were compared using the Pearson chi-square (χ²) test. All tests were two-sided, with p < 0.05 considered statistically significant. No statistically significant baseline differences were identified between the two groups. Abbreviations: PGE1, prostaglandin E1 (alprostadil); VAS, Visual Analog Scale; SD, standard deviation; χ², chi-square

Variable	Fogarty balloon-assisted flow augmentation + PGE1 (n = 50)	PGE1 alone (n = 50)	p-value
Age (years)	63.8 ± 8.4	64.5 ± 9.1	0.71
Male sex	37 (74.0%)	35 (70.0%)	0.65
Diabetes mellitus	39 (78.0%)	40 (80.0%)	0.81
Hypertension	34 (68.0%)	36 (72.0%)	0.66
Dyslipidemia	29 (58.0%)	27 (54.0%)	0.68
Current smoker	30 (60.0%)	29 (58.0%)	0.84
Chronic kidney disease	12 (24.0%)	13 (26.0%)	0.82
Coronary artery disease	18 (36.0%)	17 (34.0%)	0.83
Rutherford category 5	35 (70.0%)	34 (68.0%)	0.83
Rutherford category 6	15 (30.0%)	16 (32.0%)	0.83
Baseline VAS pain score	8.4 ± 1.0	8.2 ± 1.1	0.38

Fogarty balloon-assisted flow augmentation was technically completed in 46 of 50 patients (92.0%), while technical completion was not achieved in four patients (8.0%). The mean operative duration was 54 ± 13 minutes. One patient (2.0%) experienced minor arterial wall injury, and two patients (4.0%) developed postoperative wound hematoma. No procedure-related mortality occurred during the index hospitalization.

Technical completion was defined as successful passage of the Fogarty catheter through the intended arterial segment with satisfactory distal back bleeding or retrograde flow at completion of the procedure. This definition reflected completion of the surgical maneuver and intraoperative distal perfusion assessment and did not constitute angiographic confirmation of arterial recanalization or anatomical luminal dilatation (Table 2).

**Table 2:** Procedural outcomes of the Fogarty balloon-assisted flow-augmentation group Technical completion was defined as successful passage of the Fogarty catheter through the intended arterial segment with satisfactory distal back bleeding or retrograde flow at completion. This operational definition reflected completion of the surgical maneuver and intraoperative distal perfusion assessment and did not constitute angiographic confirmation of arterial recanalization or anatomical luminal dilatation. Data are presented as mean ± SD or number (percentage), as appropriate.

Variable	Value (n = 50)
Technical success	46 (92.0%)
Technical failure	4 (8.0%)
Mean operative time (minutes)	54 ± 13
Minor arterial wall injury	1 (2.0%)
Postoperative wound hematoma	2 (4.0%)
Procedure-related mortality	0 (0%)

At six months, limb salvage was achieved in 41 of 50 patients (82.0%) in the Fogarty balloon-assisted flow augmentation plus PGE1 group compared with 30 of 50 patients (60.0%) in the PGE1-alone group (Pearson χ² = 5.88, p = 0.015). Major amputation occurred in nine (18.0%) patients in the intervention group and 20 (40.0%) patients in the control group, representing an absolute between-group difference of 22.0 percentage points (p = 0.015).

Among patients with an ischemic ulcer at baseline, complete ulcer healing occurred in 34 of 41 patients (82.9%) in the intervention group compared with 22 of 34 patients (64.7%) in the PGE1-alone group. Although the complete ulcer-healing rate was numerically higher in the intervention group, the difference did not reach statistical significance (Pearson χ² = 3.26, p = 0.071). Improvement of at least one Rutherford clinical category occurred in 40 (80.0%) patients receiving Fogarty balloon-assisted flow augmentation plus PGE1 compared with 28 (56.0%) patients receiving PGE1 alone (Pearson χ² = 6.62, p = 0.010). Minor amputation was required in seven (14.0%) patients in the intervention group and 11 (22.0%) patients in the control group, with no statistically significant difference between groups (p = 0.30). Six-month all-cause mortality was two (4.0%) patients in the intervention group and three (6.0%) patients in the control group (p = 0.65). No additional procedure-related complications requiring reoperation were observed during follow-up (Table 3).

**Table 3:** Six-month clinical outcomes in patients with no-option below-knee chronic limb-threatening ischemia Data are presented as number (percentage). Complete ulcer healing was assessed among patients with an ischemic ulcer at baseline and was defined as complete epithelialization of the index ulcer without drainage or the need for further surgical wound intervention. Limb salvage was defined as freedom from major (above-ankle) amputation during follow-up. Major amputation was defined as amputation performed above the ankle. Between-group comparisons were performed using Pearson's chi-square test. All tests were two-sided, with p < 0.05 considered statistically significant. The difference in complete ulcer-healing rates was not statistically significant. Abbreviations: PGE1, prostaglandin E1 (alprostadil); χ², Pearson chi-square statistic

Outcome	Fogarty balloon-assisted flow augmentation + PGE1 (n = 50)	PGE1 alone (n = 50)	Test statistic	p value
Limb salvage	41 (82.0%)	30 (60.0%)	χ² = 5.88	0.015
Major amputation	9 (18.0%)	20 (40.0%)	χ² = 5.88	0.015
Complete ulcer healing*	34/41 (82.9%)	22/34 (64.7%)	χ² = 3.26	0.071
≥1 Rutherford category improvement	40 (80.0%)	28 (56.0%)	χ² = 6.62	0.010
Minor amputation	7 (14.0%)	11 (22.0%)	χ² = 1.08	0.30
Six-month all-cause mortality	2 (4.0%)	3 (6.0%)	χ² = 0.21	0.65

Both groups demonstrated progressive reductions in ischemic rest pain during follow-up. In the Fogarty balloon-assisted flow augmentation plus PGE1 group, mean VAS scores decreased from 8.4 ± 1.0 at baseline to 4.5 ± 1.3 at one month, 3.2 ± 1.4 at three months, and 2.6 ± 1.5 at six months. In the PGE1-alone group, mean VAS scores decreased from 8.2 ± 1.1 at baseline to 6.3 ± 1.5, 5.2 ± 1.6, and 4.5 ± 1.8 at one, three, and six months, respectively. The between-group difference was not statistically significant at baseline (p = 0.38), but mean VAS scores were significantly lower in the intervention group at one, three, and six months (all p < 0.001) (Table 4).

**Table 4:** Comparison of Visual Analog Scale (VAS) pain scores during the six-month follow-up VAS pain scores were recorded on a 0–10 scale, with higher scores indicating greater pain. Values are presented as mean ± standard deviation. Between-group comparisons at each prespecified follow-up time point were performed using the independent-samples Student's t-test. A two-tailed p value <0.05 was considered statistically significant. Abbreviations: VAS, Visual Analog Scale; PGE1, prostaglandin E1 (alprostadil); SD, standard deviation.

Follow-up time	Fogarty balloon-assisted flow augmentation + PGE1 (n = 50), mean ± SD	PGE1 alone (n = 50), mean ± SD	t-value	p-value
Baseline	8.4 ± 1.0	8.2 ± 1.1	0.88	0.38
1 month	4.5 ± 1.3	6.3 ± 1.5	6.43	<0.001
3 months	3.2 ± 1.4	5.2 ± 1.6	6.68	<0.001
6 months	2.6 ± 1.5	4.5 ± 1.8	5.75	<0.001

These findings indicate that patients receiving the combined intervention had lower mean ischemic pain scores at each follow-up assessment after the baseline evaluation. Because the analysis was based on between-group comparisons at each prespecified time point, no repeated-measures analysis of variance was performed.

## Discussion

This prospective quasi-experimental study evaluated the clinical outcomes of Fogarty balloon-assisted flow augmentation combined with intravenous prostaglandin E1 compared with intravenous prostaglandin E1 alone in patients with no-option below-knee CLTI managed in a resource-limited tertiary care hospital. The principal findings were that the combined treatment strategy was associated with significantly higher limb salvage, lower major amputation rates, and lower VAS pain scores, while the complete ulcer-healing rate was numerically higher but did not differ significantly between groups. The procedure was also associated with a low incidence of procedure-related complications.

Limb salvage remains the principal therapeutic objective in patients with advanced CLTI because major amputation is associated with substantial morbidity, impaired functional independence, reduced quality of life, and increased mortality. Despite advances in endovascular and surgical revascularization, patients with diffuse infrapopliteal disease who are unsuitable for bypass or endovascular intervention continue to have limited treatment options [16]. In the present study, limb salvage was achieved in 82.0% of patients treated with Fogarty balloon-assisted flow augmentation combined with prostaglandin E1 compared with 60.0% of those treated with prostaglandin E1 alone, with a corresponding reduction in major amputation from 40.0% to 18.0%. These findings suggest a potentially clinically meaningful benefit of the combined approach in carefully selected patients with no-option CLTI when conventional revascularization cannot be performed. However, given the non-randomized study design, these findings should be interpreted as an association rather than definitive evidence of treatment efficacy.

Improvement of at least one Rutherford clinical category occurred in 80.0% of patients in the intervention group compared with 56.0% in the PGE1-alone group, representing a statistically significant between-group difference (p = 0.010). This finding is clinically relevant because Rutherford category incorporates important manifestations of limb ischemia and therefore provides a clinically meaningful measure of improvement. The greater improvement observed with the combined treatment may indicate an incremental clinical benefit beyond prostaglandin therapy alone. Nevertheless, because Rutherford category is a clinical measure, the observed improvement cannot by itself establish restoration of arterial patency or a specific physiological mechanism.

Ulcer healing should be interpreted more cautiously. Among patients who had an ischemic ulcer at baseline, complete healing occurred in 34 of 41 patients (82.9%) in the intervention group compared with 22 of 34 patients (64.7%) in the PGE1-alone group. Although the absolute proportion of complete healing was approximately 18 percentage points higher with the combined treatment, the difference did not reach statistical significance (Pearson χ² = 3.26, p = 0.071). Thus, the present study provides a clinically encouraging signal for improved ulcer healing but does not establish a statistically significant treatment effect. Furthermore, ulcer healing may be influenced by wound size, depth, infection, tissue loss, nutritional status, local wound care, and anatomical distribution [17]. These factors were not sufficiently characterized to determine whether they contributed to the observed difference. Larger studies with standardized wound assessment and objective perfusion measurements are therefore required to clarify this potential benefit.

Both treatment groups demonstrated progressive improvement in ischemic rest pain during follow-up; however, the reduction in VAS scores was significantly greater among patients undergoing the Fogarty balloon-assisted flow-augmentation technique. Similarly, complete ulcer healing occurred more frequently in the intervention group. These findings are biologically plausible because prostaglandin E1 has established vasodilatory, antiplatelet, anti-inflammatory, and microcirculatory effects that improve tissue oxygenation and reduce ischemic symptoms in patients with advanced peripheral arterial disease [18,19]. Nevertheless, prostaglandin therapy alone has generally demonstrated only modest effects on limb salvage and does not restore large-vessel patency [18,19]. The improved clinical outcomes observed in the present study therefore suggest that the additional surgical procedure may have provided incremental benefit beyond pharmacological therapy alone.

Minor amputation was required in 14.0% of patients in the intervention group and 22.0% in the PGE1-alone group; however, this difference was not statistically significant (p = 0.30). Accordingly, the present study does not provide sufficient evidence that the combined treatment reduces the requirement for minor amputation. The lower observed proportion in the intervention group may nevertheless warrant further investigation in larger cohorts, particularly because preservation of the foot and avoidance of tissue loss remain clinically important goals in CLTI.

Six-month all-cause mortality was also similar between groups, occurring in 4.0% of patients in the intervention group and 6.0% of patients in the PGE1-alone group (p = 0.65). The study was not designed or powered to detect differences in mortality, and the small number of deaths limits meaningful interpretation of this outcome. Therefore, the observed mortality rates should be considered descriptive rather than evidence of a mortality benefit associated with the intervention.

One of the most important concepts supporting the rationale of this study is indirect revascularization through collateral circulation. The angiosome concept describes the lower limb as a series of three-dimensional vascular territories supplied by specific source arteries that are interconnected by arterial-arterial collateral channels, commonly referred to as choke vessels [9]. Although direct revascularization of the artery supplying the affected angiosome generally provides the highest rates of wound healing, several clinical studies have shown that satisfactory limb salvage may also be achieved through indirect revascularization when collateral networks remain functional [9-11]. We hypothesize that gentle catheter passage may be associated with changes in residual arterial flow and collateral perfusion, potentially facilitating indirect angiosomal perfusion through the existing collateral network. When combined with the microvascular vasodilatory effects of prostaglandin E1, even relatively small improvements in tissue perfusion may be sufficient to enhance ulcer healing and reduce ischemic pain. However, this proposed mechanism remains hypothetical and requires confirmation through dedicated physiological and imaging studies.

It is important to emphasize that Fogarty balloon-assisted flow augmentation should not be considered equivalent to conventional endovascular balloon angioplasty. Contemporary international guidelines continue to recommend endovascular intervention or surgical bypass whenever anatomically feasible because restoration of arterial patency remains the cornerstone of CLTI management [20]. Conventional balloon angioplasty requires guidewire crossing of the lesion, high-pressure angioplasty balloons, fluoroscopic guidance, contrast angiography, and dedicated endovascular equipment. These facilities were unavailable at our institution during the study period, precluding standard endovascular treatment. Consequently, the technique investigated in this study should be regarded as a resource-adapted surgical adjunct intended for patients with no-option CLTI rather than as an alternative to established endovascular therapy.

Equally important, the favorable outcomes observed in this study should not be interpreted as evidence that the Fogarty balloon mechanically dilates chronically occluded tibial arteries in the same manner as a high-pressure angioplasty balloon. The Fogarty catheter is a low-pressure device originally designed for arterial embolectomy and thrombectomy [15]. Therefore, the observed clinical improvements cannot be assumed to result from restoration of arterial lumen comparable to conventional angioplasty. Instead, they may reflect a combination of gentle mechanical manipulation of diseased arterial segments, optimization of residual blood flow, recruitment of collateral circulation, enhancement of choke vessel perfusion, and improved microvascular circulation mediated by prostaglandin E1. Because no routine duplex ultrasonography, computed tomography angiography, transcutaneous oxygen pressure measurement, or perfusion imaging was performed in this study, these mechanisms remain speculative and should be interpreted cautiously.

Our findings should therefore be interpreted primarily from a clinical rather than a mechanistic perspective. The study demonstrates improvements in patient-centered outcomes-including limb salvage, ischemic pain, ulcer healing, and major amputation-in a carefully selected population of patients with no-option CLTI managed in a resource-constrained environment. Whether these benefits result predominantly from changes in macrovascular flow, collateral recruitment, microvascular perfusion, or a combination of these mechanisms remains uncertain and warrants further investigation.

The present findings are broadly consistent with previous studies demonstrating that prostaglandin E1 improves ischemic pain, tissue perfusion, and ulcer healing in patients with advanced peripheral arterial disease, although previous studies have generally shown only limited reductions in major amputation rates [18,19]. Contemporary guidelines emphasize revascularization when feasible and note that the effectiveness of prostanoid therapy in no-option CLTI remains uncertain; therefore, the present findings should be viewed as preliminary evidence regarding an exploratory adjunctive surgical approach rather than evidence establishing a new standard of care [3,14,21,22]. Accordingly, our results should not be interpreted as challenging the established role of endovascular or surgical revascularization. Rather, they suggest that a simple, inexpensive surgical adjunct may merit further investigation in healthcare systems where modern endovascular facilities are unavailable or unaffordable.

The procedure also demonstrated an acceptable safety profile. Technical success was achieved in 92% of patients, while only one minor arterial wall injury and two postoperative wound hematomas were observed. No procedure-related mortality occurred. Although these findings suggest that the technique can be performed safely by experienced vascular surgeons, confirmation of its reproducibility will require evaluation across multiple centers and by independent investigators.

Several strengths of this study deserve mention. The investigation was prospective, consecutive patients were enrolled using predefined eligibility criteria, complete six-month follow-up was achieved, and clinically meaningful endpoints-including limb salvage, major amputation, ulcer healing, and pain reduction-were systematically assessed. Furthermore, this study addresses a clinically important problem frequently encountered in low- and middle-income countries, where advanced endovascular technologies are often unavailable and therapeutic options for patients with no-option CLTI remain extremely limited.

Several limitations should also be acknowledged. First, the quasi-experimental design without randomization introduces the possibility of selection bias despite comparable baseline characteristics between treatment groups. Second, treatment allocation depended on surgeon availability, operating room access, and patient preference rather than random assignment. Third, the study was conducted at a single center with a relatively small sample size, limiting external validity. Fourth, the follow-up period of six months was insufficient to evaluate long-term durability, restenosis, recurrent ischemia, or late limb salvage. Fifth, objective vascular imaging and physiological perfusion assessments were not routinely performed because of resource limitations, preventing confirmation of the proposed mechanism of action. Finally, because this represents the first clinical evaluation of Fogarty balloon-assisted flow augmentation, the findings should be considered exploratory and hypothesis-generating rather than definitive evidence of efficacy.

Future research should include multicenter prospective studies with larger patient populations, longer follow-up, objective perfusion assessment using duplex ultrasonography, computed tomography angiography, transcutaneous oxygen pressure measurement, and perfusion imaging, as well as randomized comparisons with contemporary treatment strategies where feasible. Such studies are necessary to determine the reproducibility, mechanism of action, long-term effectiveness, and appropriate clinical role of this novel surgical technique.

Overall, the present study suggests that Fogarty balloon-assisted flow augmentation combined with intravenous prostaglandin E1 may represent a feasible limb-salvage strategy for carefully selected patients with no-option below-knee CLTI in resource-limited settings where conventional endovascular intervention or distal bypass surgery is unavailable or technically infeasible. However, this technique should not be considered a replacement for established endovascular balloon angioplasty or surgical bypass, both of which remain the preferred treatment options whenever anatomically feasible. Instead, these findings provide preliminary clinical evidence supporting further investigation of this resource-appropriate surgical adjunct in patients who otherwise have very limited therapeutic alternatives.

## Conclusions

In this prospective, single-center quasi-experimental study of patients with no-option below-knee CLTI, Fogarty balloon-assisted flow augmentation combined with intravenous PGE1 was associated with higher six-month limb salvage, lower major amputation rates, and greater reduction in ischemic rest pain compared with PGE1 alone. The complete ulcer-healing rate was numerically higher in the combined-treatment group, although this difference did not reach statistical significance. These findings should be interpreted cautiously because treatment allocation was non-randomized, the study was conducted at a single resource-limited center, and objective post-procedural perfusion and anatomical recanalization were not routinely assessed. The technique should therefore be regarded as an exploratory salvage approach rather than a substitute for conventional endovascular or surgical revascularization. Larger, adequately controlled studies with objective perfusion assessment and longer follow-up are required.
